# Case 5/2019 - 55-Year-Old Diabetic Man with Heart Failure After Non-ST Segment Elevation Myocardial Infarction

**DOI:** 10.5935/abc.20190225

**Published:** 2019-10

**Authors:** Ana Vitória Vitoreti Martins, José Roberto de Oliveira da Silva, Paulo Sampaio Gutierrez

**Affiliations:** Instituto do Coração (InCor) HC-FMUSP, São Paulo, SP - Brazil

**Keywords:** Diabetes Mellitus/complications, ST- Elevation Myocardial Infarction, Heart Failure, Cardiogenic Schock

A 55-year-old male patient which is insulin-dependent diabetes mellitus (DM) with visual complications and chronic kidney disease (CKD) was admitted a month ago (February 2017) after an episode of nocturnal dyspnea. A diagnosis of non-ST-segment elevation myocardial infarction (AMI) was identified. Admission examinations revealed elevated myocardial injury markers - CKMB of 70 ng/mL and 2 ng/mL troponin. The electrocardiogram (ECG) showed a ST-segment depression from _V2_ to _V6_. The coronary angiography revealed a 30% lesion in the right and left coronary arteries did not present lesions (Figure 1). The echo revealed diffuse left ventricular hypokinesia and 36% ejection fraction. Creatinine was 1.8 mg/dL.

**Figure 1:**
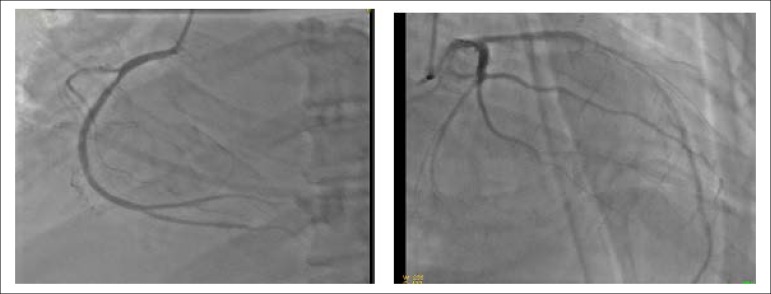
Right coronary in right anterior oblique view. A) Left coronary in right oblique view (B)

After discharge, the patient developed orthopnea and lower limb edema and sought emergency care and was transferred to InCor two weeks after hospital discharge with a diagnosis of decompensated heart failure (March 22, 2017).

During a physical examination, the patient had dyspnea, was hydrated, did not have a fever, had good peripheral perfusion, blood pressure was 100x70 mmHg, a 90 bpm heart rate and 92% oxygen saturation. There was jugular turgescence, bilateral and symmetrical vesicular murmur present, presence of crackles in both lung bases, normophonetic rhythmic sounds in two stages, without murmurs. There was no hepatomegaly or hepatojugular reflux. The patient's abdomen was flaccid, painless and had airborne noises present. The lower limbs presented a ++ / 4+ edema, the calves were free and there were symmetrical pedis pulses.

The patient was using 100mg acetylsalicylic acid, 20 mg enalapril and NPH human insulin.

The ECG (March 22, 2017) revealed sinus rhythm, low voltage of the QRS complex in the frontal plane and ST segment depression, 1 mm, horizontal from V_2_ to V5, and a reduction of the left ventricular potentials (Figure 2).

**Figure 2:**
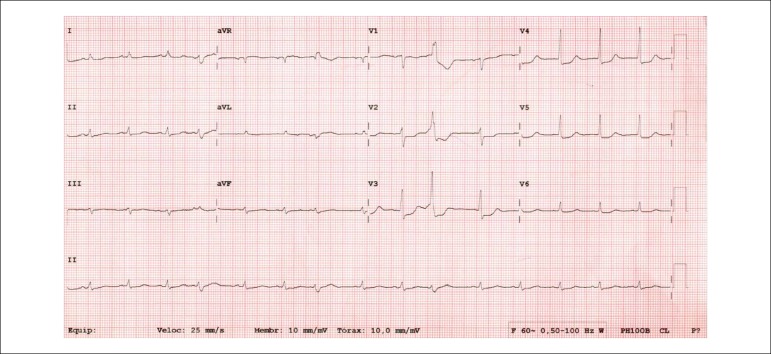
Low-voltage electrocardiogram of the QRS complex in the frontal plane and ST-segment depression from V_2_ to V_5_.

Chest radiography (March 23, 2017) revealed massive bilateral pleural effusion and cardiomegaly (Figure 3), persistently identified on the radiograph on March 31, 2017.

**Figure 3:**
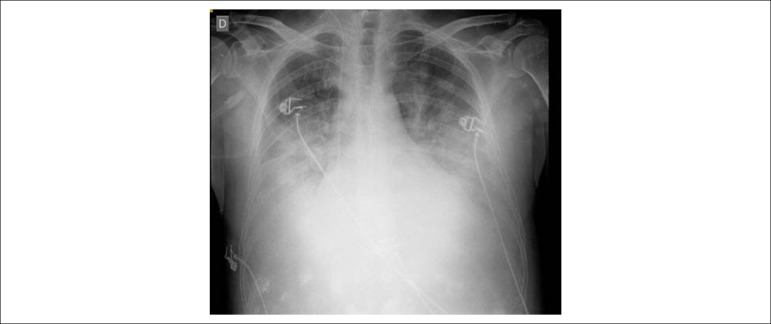
Chest X-ray: massive bilateral pleural effusion and cardiomegaly.

Laboratory tests revealed anemia and creatinine increases (Table 1).

**Table 1:** Laboratory Exams

Exams	03/23/2017	03/27/2017	03/30/2017	03/31/2017
Hemoglobin, g/dL	11.3	9.4	9.3	8.5
Hematocrit (%)	35	29	27	25
Leukocytes (/mm^3^)	9600	16000	9470	10030
Rod cells (%)	n	8	6	
Segmented	n	81	80	
Neutrophils (%)	67	89	86	81
Eosinophils:	2	0	0	0
Basophils (%)	0	0	0	0
Lymphocytes (%)	21	5	9	13
Monocytes (%)	10	6	3	6
Platelets/mm^3^	202000	151000	180000	129000
CKMB (ng/mL)	14.5			
Troponin I (ng/mL)	3.65			
Calcium (mg/dL)	8.5		8.5	
Ionic calcium (mMol/L)	1.24			
Phosphorus (mg/dL)			5.1	5.1
Magnesium (mg/dL)	1.8	1.8	2.3	2.4
PCR (mg/L)	3.13	91.39	53.16	48.25
Sodium (mEq/L)	139		138	
Potassium (mEq/L)	4.4		3.2	
Urea (mg/dl)	56	66	131	144
Creatinine(mg/dl)	2.34	2.62	3.46	4.01
Gasometry		venous	artery	
pH		7,36	6.92	
pCO_2_ (mmHg)		43.2	56.1	
pO_2_ (mmHg)		39.7	59.1	
O_2_ saturation (%)		63.2	11	
HCO_3_- (mEq/L)		23.9	-22.9	
BE (mEq/l)		-1		
tAP (INR)			2.5	
TTPA (rel)			1.22	
Dimer D (ng/mL)			704	
Fibrinogen (mg/L)			327	
Arterial lactate (mg/dL)		24	134	
AST (U/L)		33		46
ALT (U/L)		37		35
Lactic Dehydrogenase (U/L)				293
Total bilirubins (mg/dL)		0.45		0.67
Direct bilirubin (mg/dL)		0.23		0.34

CKMB: creatinokinase MB; PCR: C reactive protein; BE: base excess; tAP (INR): prothrombin time; TTPA: Partial thromboplastin time; AST: aspartate aminotransferase; ALT: alanine transaminase

Echocardiography (March 23, 2017) revealed a 30 mm aortic diameter, 39mm left atrium, right ventricle in the basal portion, which was 44 mm and in the middle portion, which was 29 mm. The septum and posterior wall thickness were 7 mm, left ventricular diameters of 56 mm in diastole and 51 mm in systole, the area method ejection fraction (Simpson) was 22%. There was mild to moderate mitral regurgitation and pulmonary artery systolic pressure was 36 mmHg.

The serologies for Chagas disease and cytomegalovirus were negative.

With this history and the exam findings, the hypothesis of type 2 infarction or myocarditis was raised.

Magnetic nuclear resonance showed marked biventricular systolic dysfunction - 11% left ventricular ejection fraction (LVEF) and slight dilatation (indexed final diastolic volume of 106 mL/m^2^ and final systolic volume of 100 mL/m^2^), with increased right ventricular dilation (final diastolic volume indexed was 137 mL/m^2^ and final systolic volume of 122 mL/m², 6% ejection fraction). The right atrium had a normal volume while the left atrium was greatly enlarged (indexed volume 65 mL/m^2^).

The cardiac diameters were: aorta 2.5 mm, right ventricle in the longest axis 71 mm and shortest axis 40 mm; left ventricle diastole 57 mm, systole 56 mm; septum thickness 6 mm and 5mm lateral wall. There was a transmural, circumferential multifocal late enhancement, sparing the apical segments beyond the apex and compromising the papillary muscles, which all had subendocardium involvement (Figure 4). There was no pericardial effusion and there was massive bilateral pleural effusion.

**Figure 4:**
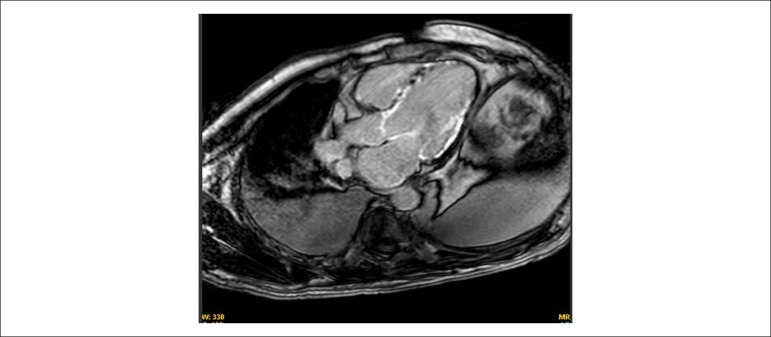
4-chamber magnetic resonance imaging - circumferential late enhancement.

During hospitalization, the patient had a fever and leukocytosis (Table 1), and was diagnosed with bronchopneumonia, treated with vancomycin and tazobactam with a reduction in C-reactive protein and leukocytosis.

Renal function worsened (Table 1) with oliguria and a shilley catheter was used in the femoral vein, but due to hemodynamic instability, hemodialysis was not possible.

Since admission, the patient had severe dyspnea requiring long-term non-invasive ventilation and right hemithorax drainage due to respiratory discomfort with a serohematic discharge of about 3 liters during two days. The March 31 radiograph revealed massive bilateral pleural effusion prior to drainage. In the middle of the night on March 31st, 2017, the patient had cardiopulmonary arrest with pulseless electrical activity and was promptly resuscitated for 15 minutes with a return to spontaneous circulation. The patient received orotracheal intubation and required maximum doses of noradrenaline and dobutamine to maintain a mean blood pressure of 65 mmHg. He evolved with a refractory shock, with mechanical ventilation difficulties and died at 12:05 (March 31st, 2017).

## Clinical Aspects

This is a 55-year-old male patient known to have insulin dependent DM and CKD who in February 2017 had a nocturnal episode of dyspnea. Due to the episode, he sought medical care in the emergency room, where changes in markers of myocardial necrosis were identified - increased CKMB and troponin, as well as ST-segment depression from V_2_ to V_6_ on the ECG. A coronary angiography was performed at the time showing only a 30% lesion in the right coronary artery.

After discharge, the patient developed orthopnea and lower limb edema requiring further hospitalization seven days after discharge. On admission to our service, he presented signs of pulmonary and systemic congestion, the ECG maintained previous parameters, a chest X-ray showed cardiomegaly and massive bilateral pleural effusion, and laboratory tests showed a worsening of renal function and anemia. The transthoracic echocardiogram showed 22% LVEF, through the Simpson method, with diffuse hypokinesia and no segment changes. After this initial evaluation, the following hypothesis was raised for this patient - type 2 AMI and myocarditis. In order to enable a better investigation of the patient´s conditions, propaedeutic complementation was performed. The serologies for Chagas disease and cytomegalovirus were negative. A cardiac resonance showed increased systolic dysfunction in both ventricles with 11% LVEF, with slight left ventricular dilation, increased right ventricular dilation, a significant enlargement of the left atrium and an absence of changes in the right atrium. Regarding the enhancement, the patient presented late transmural, circumferential multifocal enhancement, with subendocardial involvement sparing the apex. There was no pericardial effusion, but there was massive bilateral pleural effusion.

During hospitalization, the patient developed pulmonary focal sepsis, with an initial improvement after the introduction of an antimicrobial regimen. Due to the need for infectious treatment, the team decided to postpone endomyocardial biopsy. Despite the improvement of infectious parameters, there was a worsening in renal function and hemodynamic instability. During the entire hospitalization period, the patient maintained a borderline respiratory pattern requiring noninvasive ventilation and right hemithorax drainage to control respiratory symptoms with serohematic secretion drainage. The patient evolved with pulseless electrical activity, reversed after resuscitation for 15 minutes. However, the patient evolved with a refractory shock, which led the patient to die 12 hours after cardiopulmonary arrest.

Due to the diagnostic doubts concerning the case and a lack of improvement after the selected therapy to control the condition, the patient was referred for autopsy with the intention of elucidating the case.

Regarding the clinical evaluation of the case, the main diagnostic hypotheses raised were myocardial infarction and myocarditis, due to the clinical presentation of acute onset heart failure with left ventricular dysfunction. The changes found on magnetic resonance imaging, although not typical, reinforced the maintenance of the initial hypotheses. We describe below information about the two clinical entities evaluated in this clinical case.

According to the fourth universal definition of MI, it consists in an increase in troponin above the 99% associated with at least one of the other factors (typical ischemic symptoms and/or new ECG abnormalities and/or imaging showing a myocardial loss with a pattern of coronary ischemia and/or thrombosis evidenced during the catheterization or autopsy).^1^ Myocardial injury does not include the changes described above and can occur in very common events in practice such as: decompensated heart failure, chronic renal failure, shock, anemia, stroke, myocarditis, Takotsubo cardiomyopathy, among others.^1^ Among the five types of AMI, type 2 occurs in the presence of an imbalance between oxygen supply and demand, in the absence of atherosclerotic plaque complications. The threshold for this imbalance to occur varies between individuals and is influenced by ongoing stressors, comorbidities (including cardiac and noncardiac), and pre-existing coronary disease.^1^ The mechanisms that influence the aforementioned imbalance are diverse and may occur concomitantly, besides being related to atherosclerosis with a reduction of myocardial perfusion and without plaque rupture, coronary spasm, microvascular dysfunction, coronary embolism, coronary dissection, tachyarrhythmias, bradyarrhythmias, hypoxemia, significant anemia, shock.^1^

The prevalence of type 2 AMI is variable in the studies and depends on the type of criteria used.^1^ In a real-life study conducted in Sweden with 20,138 patients, 7.1% of AMI hospitalizations were type 2. Patients in this group were older, predominantly female, and had more comorbidities, especially heart failure and atrial fibrillation.^2^ In a 2016 study, the main causes of type 2 AMI were - tachyarrhythmias in 36.7%; aortic stenosis in 14.5% and heart failure in 13.7%.^3^ Another study observed that approximately 50% of the patients with type 2 AMI had no significant coronary disease.^4^

Regarding the initial evaluation of these patients, the most common symptom presented was dyspnea.^3^ In addition, ST segment elevation is known to occur in 3-24% of cases. Coronary atherosclerosis is a common angiographic finding among these patients and, in general, they have a worse prognosis.^1^ Please note that angiography is not necessary to establish the diagnosis of type 2 infarction.^1^

The long-term consequences of type 2 AMI are poorly understood. A study published in 2018 evaluated and compared outcomes of patients diagnosed with type 1 AMI, type 2 AMI, and myocardial injury and showed that the risk of death was higher among those with a history of type 2 AMI compared to those with a history of type 1 AMI, even after variable adjustment. Most deaths in the first 2 groups were due to noncardiac causes. Regarding major cardiovascular events, there was no difference between the groups. Coronary artery disease was an independent predictor of major cardiovascular events among patients with type 2 AMI or myocardial injury with a 1.71 odds ratio and a confidence index of 1.31-2.24.^5^

Management of type 2 AMI remains uncertain and there are no well-defined clinical management strategies. Initial management should be performed by controlling the precipitating factor that leads to an imbalance in the demand and supply.^6^

Another study published in 2019 showed that almost 30% of the patients in the sample were diagnosed with type 1 AMI when they actually had a type 2 AMI diagnosis.^6^

Regarding myocarditis, it is known that this entity consists of a myocardial inflammatory process with multiple presentation aspects, from asymptomatic presentations to sudden death, including heart failure and fulminant presentations in this spectrum. The etiological agents are diverse, including viral or bacterial, fungal or protozoal infections, hypersensitivity reaction, autoimmune diseases and toxins.^7^ Regarding the epidemiological evaluation, it is known that it is an underdiagnosed disease, with a bimodal peak (ranging from 1 year and 20 years) and corresponds to an important cause of cell loss by direct action necrosis of the virus, cytotoxic agents with inflammatory mediators and oxidative stress products.^8^ After the initial action process of the immune system, there may be an improvement in conditions, fighting the myocyte aggressive organism and reducing the immune response or persistence of the injury due to persistent aggressive mechanisms or exacerbated immune responses.^9^

In regards to complementary exams, there is usually an increase in inflammatory markers and there may be an increase in myocardial necrosis markers. As for ECG, there may not be changes or changes that are not specific, and the echocardiogram findings may also be variable and include diffuse hypokinesia, pericardial effusion, or segment changes. Magnetic resonance imaging is a fundamental exam, as it presents high sensitivity and specificity for myocardial inflammatory processes, and may show segment changes, regional hypertrophy, dilatation of the cardiac chambers. In the endomyocardial biopsy assessment, the exam has high levels of specificity, with moderate sensitivity. Class I indications by the Brazilian Myocarditis Guideline for biopsy include the IC level for cases lasting up to 2 weeks with no established cause with progressive haemodynamic worsening, and IC for cases beginning less than 3 months ago and more than 15 days after, no definite cause and presenting ventricular arrhythmias or second and third degree atrioventricular blocks.^10^

Regarding treatment, uncomplicated cases do not require specific approaches, only symptomatic management and rest guidelines. Heart failure cases that present decreased ejection fraction require special attention and must receive medications that are known to reduce mortality. Cases requiring antiviral treatment and immunosuppressive therapy need to be evaluated.^10^ The prognosis of myocarditis is favorable in most cases. A study published in 2019 showed that 13% of the evaluated patients had a poor evolution.^11^ Summarizing, despite raising other hypotheses for the recent onset of IC, the hypothesis of coronary disease is still the main one for this patient due to risk factors, epidemiological context and alteration of complementary exams. **(Dr. Ana Vitória Vitoreti Martins and Dr. José Roberto de Oliveira da Silva Filho)**

**Diagnosis hypothesis:** Type 2 infarction with heart failure; death from cardiogenic shock. **(Dr. Ana Vitória Vitoreti Martins and Dr. José Roberto de Oliveira da Silva Filho)**

## Necropsy

**Figure 5:**
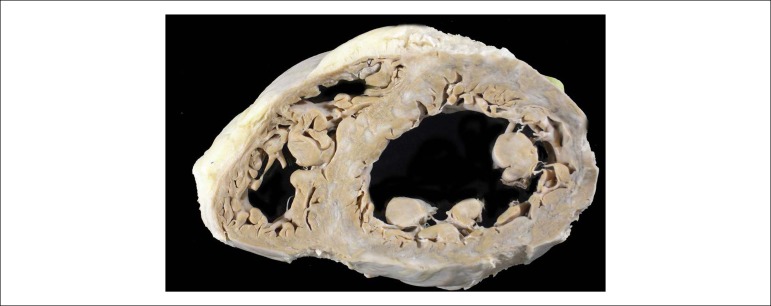
Cross section of the heart in the middle portion of the ventricles, showing grayish-white areas that correspond to end-stage infarcts in all left ventricular walls.

**Figure 6:**
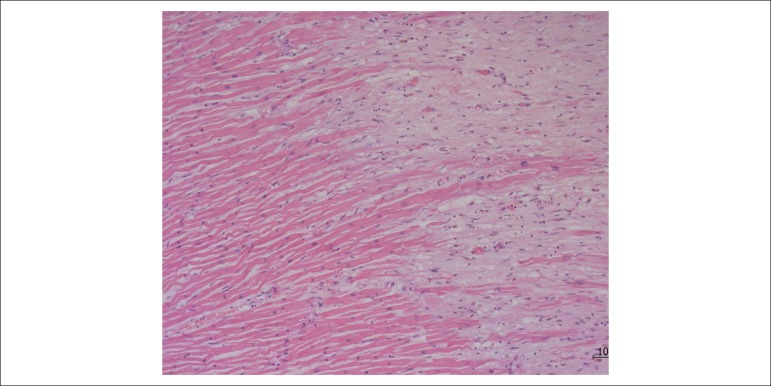
Histological section of the left ventricular myocardium showing the boundary between preserved (more rosy, left) myocardium and healing necrosis area. Hematoxylin and eosin coloring; lens magnification: 10x.

The necropsy showed the presence of myocardial infarction in the final healing phase in all left ventricular walls (Figures 5 and 6).

**Figure 7:**
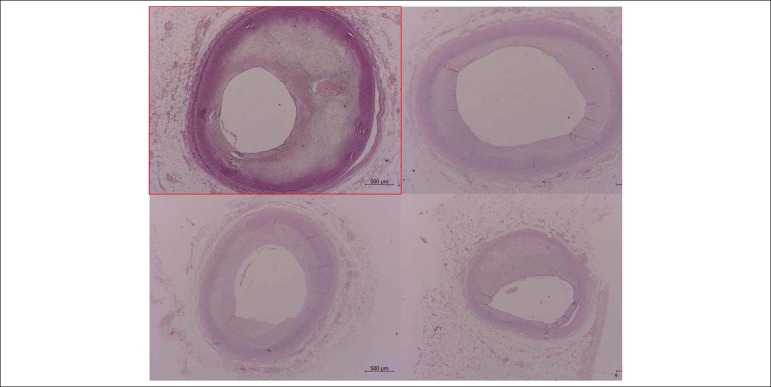
Histological sections of coronary artery segments showing moderate and focally severe atherosclerosis (4th centimeter of right coronary artery, 77% obstruction). Right coronary artery CD; Cx: circumflex branch; IVA: anterior interventricular branch (anterior descending); PVI: posterior interventricular branch (posterior descending). Verhoeff coloring for elastic fibers (right coronary artery segment) or hematoxylin and eosin (other segments); lens magnification: 2.5x.

The coronary arteries had atherosclerosis, moderate in the anterior interventricular (anterior descending) and circumflex branches of the left coronary artery (66% maximal obstructions in the first centimeter and 59% in the third centimeter, respectively) and specifically severe in the right coronary artery (77% obstruction in the fourth centimeter). There was no thrombosis or other occlusive lesions (Figure 7).

As the patient had cardiogenic shock, there was a small occipital cerebral infarction with a few days of evolution.

No cavitary lesion or thrombus occluding the left ventricular apex was evidenced.

Lung sections did not show recent bronchopneumonia.

**Figure 8:**
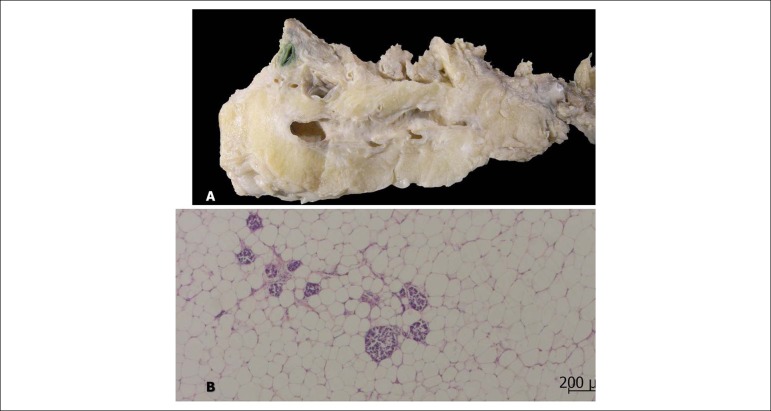
Pancreas seen both macroscopically (A) and histologically sectioned (B), hematoxylin and eosin staining, (2.5x objective enlargement) with lipomatosis, replacement of exocrine glands with fatty tissue, with only a few Langerhans islets (endocrine pancreas) left.

An important finding was pancreatic lipomatosis, with almost complete replacement of exocrine pancreatic tissue by fat, leaving only the islets (Figure 8). **Dr. Paulo Sampaio Gutierrez)**

**Anatomopathological diagnoses:** Ischemic heart disease, with myocardial infarction in the final stage of healing in all left ventricular walls. Pancreatic lipomatosis. **Dr. Paulo Sampaio Gutierrez)**

**“Causa mortis”:** cardiogenic shock **(Dr. Paulo Sampaio Gutierrez)**

## Comment

This is a very unusual case, in which some points were not clarified in the necropsy. The main point concerns the fact that the patient had a lesion with microscopic appearance of a myocardial infarction with an evolution of 4 to 6 weeks, that is, compatible with the clinical history of a sudden onset heart failure, diagnosed with infarction and elevated necrosis. However, the pattern of the lesion was not usual, irregularly affecting all the walls of the left ventricle. Therefore, we came to think of myocarditis and there was no adequate explanation for the occurrence of this ischemic necrosis; Atherosclerosis was only moderate, with a single segment with severe right coronary artery injury, and there were no recent or organizing thrombi. Diabetic patients sometimes have cardiac microcirculatory lesions, but in the present case these were not significant.

Another issue is that the pancreas had its exocrine portion almost completely replaced by fat, with the Langerhans islets left. There are three diagnoses to consider: cystic fibrosis, Schwachman-Diamond syndrome, and carboxyl lipase ester mutations. The former is eliminated due to the absence of cysts, whether in the pancreas, lungs or other organs. Schwachman-Diamond syndrome mainly affects young children. Therefore, the patient will most likely carry a carboxyl lipase ester mutation, which may even be responsible for delayed onset juvenile diabetes, as was the case with this patient, and influence the development of atherosclerosis.^12-15^**(Dr. Paulo Sampaio Gutierrez)**
